# Indirubin-pregnane X receptor-JNK axis accelerates skin wound healing

**DOI:** 10.1038/s41598-019-54754-2

**Published:** 2019-12-03

**Authors:** Yuka Tanaka, Hiroshi Uchi, Takamichi Ito, Masutaka Furue

**Affiliations:** 1 Department of Dermatology, Graduate School of Medical Sciences, Kyushu University, Fukuoka, 812-8582 Japan; 2 Department of Dermatology, National Hospital Organization Kyushu Cancer Center, Fukuoka, 811-1395 Japan; 3 Research and Clinical Center for Yusho and Dioxin, Kyushu University Hospital, Fukuoka, 812-8582 Japan; 4 Division of Skin Surface Sensing, Department of Dermatology, Graduate School of Medical Sciences, Kyushu University, Fukuoka, 812-8582 Japan

**Keywords:** Cell migration, Nuclear receptors

## Abstract

Indirubin is a potent anti-inflammatory phytochemical derived from indigo naturalis. It is also endogenously produced in the intestine and detected in the circulation in mammals. Indirubin exerts its biological functions via two xenobiotic receptor systems: aryl hydrocarbon receptor (AHR) and pregnane X receptor (PXR); however, its effects on wound healing remain elusive. To investigate whether indirubin promotes wound healing, we utilized an *in vitro* scratch injury assay and *in vivo* full-thickness mouse skin ulcer model and assessed wound closure. Indirubin significantly accelerated wound closure in both the scratch assay and the skin ulcer model. Using inhibitors of cell proliferation or migration, indirubin was found to upregulate the migratory but not the proliferative capacity of keratinocytes. Activation of AHR/PXR by indirubin was confirmed by their nuclear translocation and subsequent upregulation of *CYP1A1* (AHR), or *UGT1A1* mRNA (PXR) and also by luciferase reporter assay (PXR). Although both AHR and PXR were activated by indirubin, its pro-migratory capacity was canceled by PXR inhibition but not by AHR inhibition and was dependent on the JNK pathway. Moreover, activated PXR was detected in the nuclei of re-epithelialized keratinocytes in human skin ulcers. In conclusion, this study shows that the indirubin-PXR-JNK pathway promotes skin wound healing.

## Introduction

Indigo naturalis (Sei Tai in Japanese or Qing Dai in Chinese), prepared from leaves of plants such as *Baphicacanthus cusia*, *Polygonum tinctorium*, *Isatis indigotica*, and *Indigofera tinctoria*, has long been used as a traditional herbal drug to treat inflammatory and leukemic disorders^1,2^, and skin disorders such as eczema, aphthae, eruptions, furuncles, and psoriasis^3^. Indirubin, a stable structural isomer of indigo dye, was identified as an active component of indigo naturalis and its biological function has been confirmed in various experimental settings^1,2,4,5^. In the skin, indirubin was shown to attenuate psoriasis-like skin lesions in imiquimod-induced and Stat3-transgenic mouse models^6,7^. Topical application of indirubin also improved human psoriasis in a clinical trial without causing serious adverse events^8,9^. Indirubin is known to exert its biological function via xenobiotic aryl hydrocarbon receptor (AHR)^10^. The binding of indirubin to AHR induces its cytoplasmic-to-nuclear translocation and upregulates the transcription of downstream genes such as cytochrome P450 1A1 (*CYP1A1*)^11^.

Previous studies suggested that indirubin is produced endogenously in the human body by intestinal microbiota^12–15^. Endogenous indirubin has been detected in fetal bovine serum (FBS) and even in human urine^10^. In FBS, the concentration of indirubin accounts for almost half of the total AHR activity^10^. In keratinocytes, the AHR activation by indirubin inhibits cell proliferation and induces terminal differentiation by upregulating involucrin, which may contribute to some of its efficacy on psoriasis^3,7^.

AHR is also negatively involved in wound healing. In mice deficient in *Ahr*, wound healing is markedly accelerated^16^. Lack of *Ahr* increases keratinocyte migration and accelerates skin re-epithelialization without affecting cell proliferation or the recruitment of inflammatory cells^16^. Since indirubin is a potent AHR activator^10^, it is expected to potentially inhibit wound healing. However, conflicting evidence has suggested that indirubin enhances intestinal epithelial wound healing through the activation of another xenobiotic receptor, pregnane X receptor (PXR, also known as nuclear receptor subfamily 1 group I member 2, NR1I2)^17,18^. PXR is one of the nuclear receptors and ligand-activated transcription factors, which acts as another general sensor of xenobiotics^19–21^. PXR can be activated by a wide range of xenobiotics and chemicals, such as steroid, retinoid, bile acid, and rifampicin, because of its unique flexible ligand binding pocket^22^. Indirubin activates PXR and upregulates the expression of its downstream responsive genes, such as *CYP3A4* (a potent xenobiotic-catabolizing enzyme^17,20^) and UDT glucuronosyltransferase family 1 member A1 (*UGT1A1*, an enzyme of the glucuronidation pathway^23,24^). To the best of our knowledge, the effect of indirubin on cutaneous wound healing remains elusive. In this study, we demonstrate that indirubin is a dual activator of AHR and PXR in human keratinocytes. Indirubin accelerates keratinocyte wound healing via PXR, but not AHR.

## Results

### Indirubin accelerates wound closure both *in vitro* and *in vivo*

**Figure 1:**
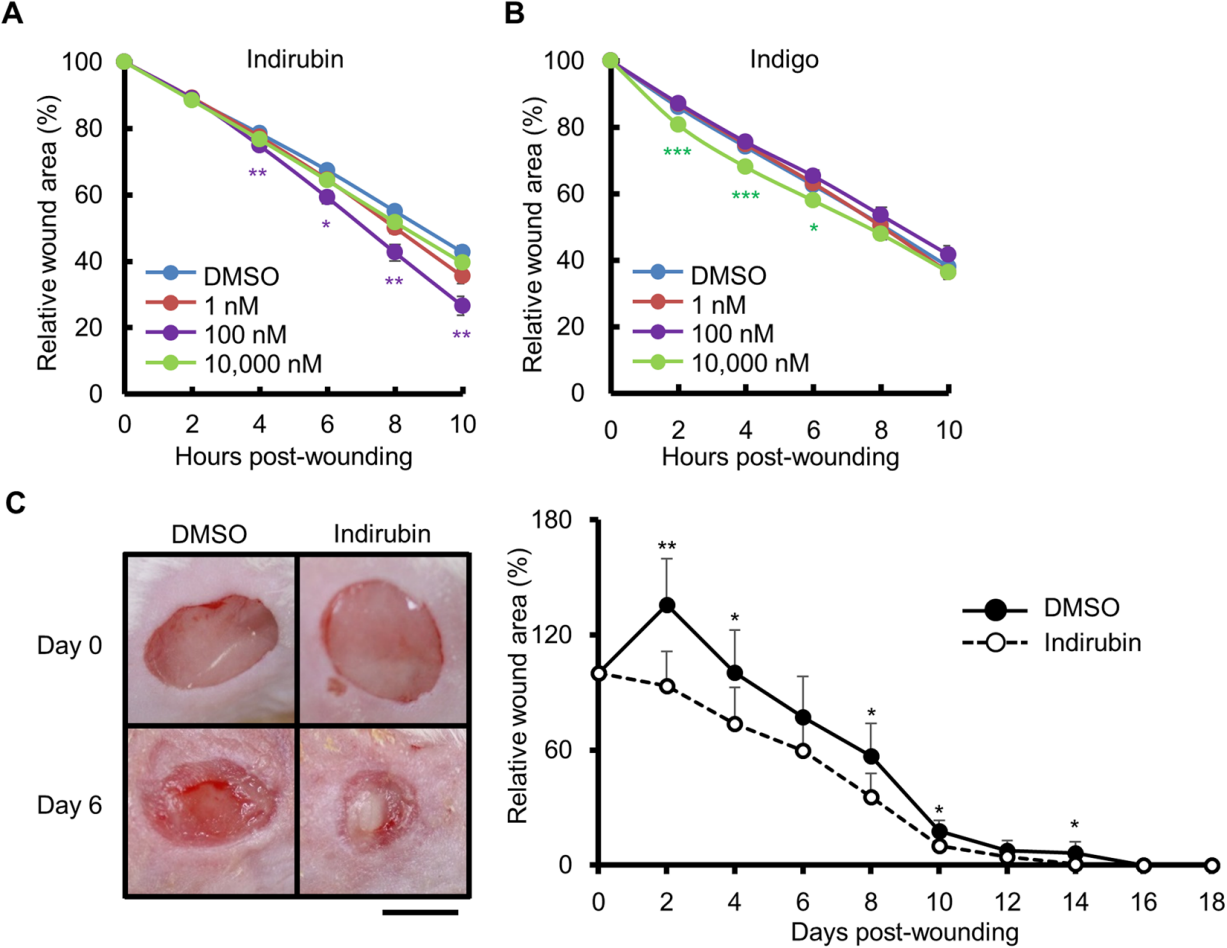
Indirubin promotes wound healing both *in vitro* and *in vivo*. (**A**) Scratched cells were treated with DMSO (0.1%) or indirubin (1, 100, or 10,000 nM) and the relative wound areas were measured (n = 18). (**B**) Scratched cells were treated with DMSO (0.1%) or indigo (1, 100, or 10,000 nM) and the relative wound areas were measured (n = 18). (**C**) Full-thickness wounds were created on the dorsal skin of BALB/c mice, which were then treated with Vaseline containing DMSO (1%) or indirubin (262.26 ng/g Vaseline). Representative images (left) and the relative wound areas (right) after wound creation are shown (n = 6). Scale bar = 5 mm. All data are presented as mean ± SD. *P < 0.05, **P < 0.01, and ***P < 0.001.

Indirubin did not show any cytotoxicity at concentrations up to 10 μM in HaCaT keratinocytes (Supplementary Fig. S1). We then examined its effect on wound healing using an *in vitro* scratch assay. The areas of wounds were reduced significantly more rapidly upon treatment with indirubin (100 nM) than upon treatment with DMSO (Fig. 1A). We also assessed the effect of indigo, which is a structural isomer of indirubin. Although indigo and indirubin have similar structures, the wound-healing effect of indigo was transient and only occurred during 2 to 6 h after wounding in the scratch assay (Fig. 1B), and was only observed at a higher concentration (10 μM) than that of indirubin (100 nM).

Since indirubin promoted wound closure in keratinocytes, we evaluated its effect *in vivo* using mice with full-thickness wounds on their dorsal skin. When ointment containing indirubin was applied to the wounds, it significantly promoted wound closure compared with that in vehicle (DMSO)-treated mice (Fig. 1C).

### Indirubin promotes keratinocyte migration, but not proliferation

**Figure 2:**
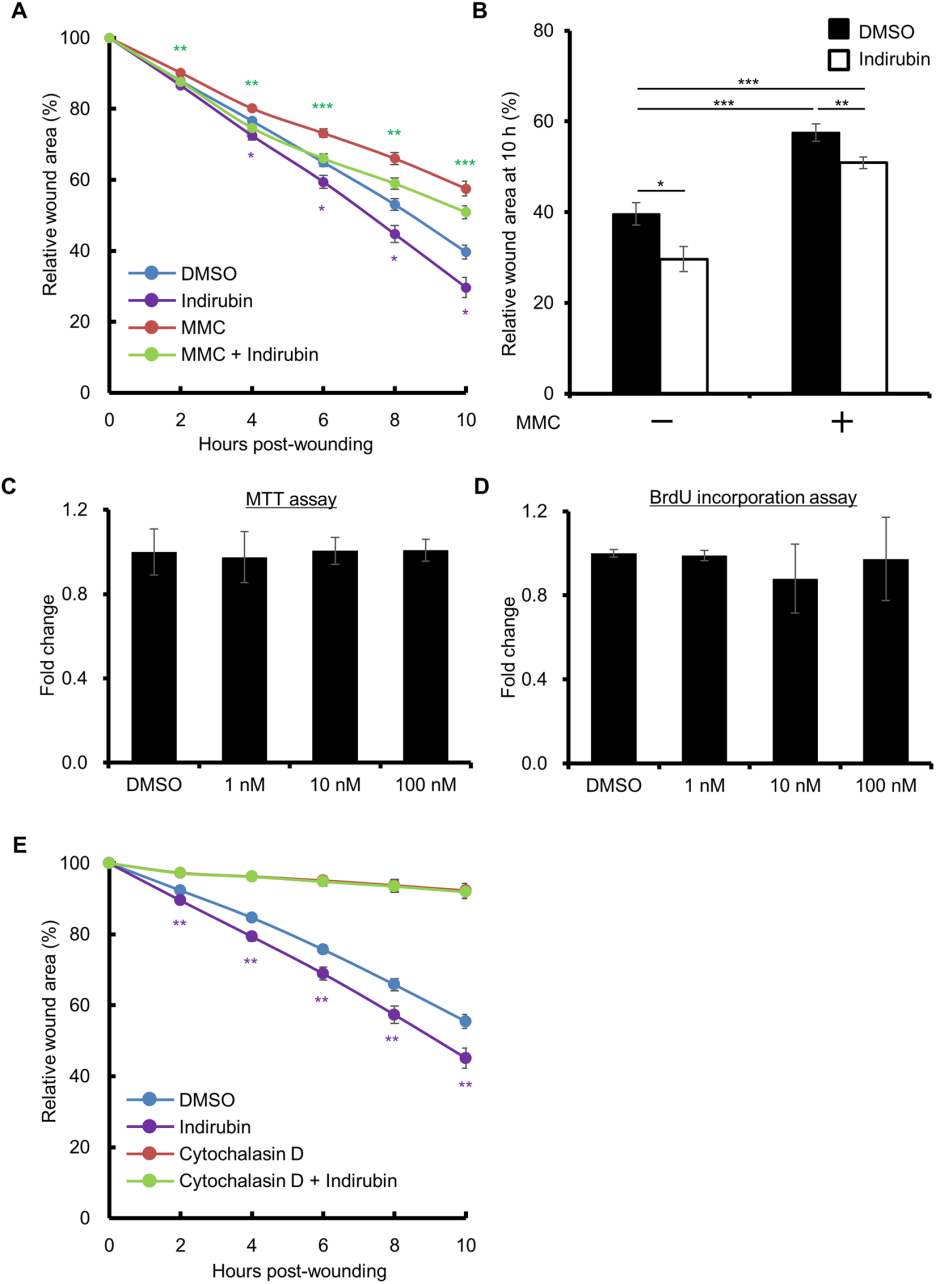
Indirubin promotes migration of keratinocytes, but not their proliferation. (**A**) HaCaT cells were treated without or with mitomycin C (MMC, 5 μg/mL) for 2 h, scratched, and treated with DMSO (0.1%) or indirubin (100 nM). Relative wound areas are shown (n = 18). (**B**) The wound area at 10 h post-wounding relative to that of (**A**) is shown. (**C**,**D**) HaCaT cells were treated with indirubin (1, 10, or 100 nM) for 24 h and were assessed for cell proliferation using (**C**) MTT assay or (**D**) BrdU incorporation assay (n = 6). (**E**) HaCaT cells were scratched and treated with DMSO (0.1%) or indirubin (100 nM) in the absence or presence of cytochalasin D (2 μM). Relative wound areas are shown (n = 18). All data are presented as mean ± SD. *P < 0.05, **P < 0.01, and ***P < 0.001.

We next aimed to elucidate how indirubin promotes wound closure. There are two ways in which this can be achieved: acceleration of cell proliferation and promotion of cell migration. As shown in Fig. 2A,B, inhibition of cell proliferation by mitomycin C (MMC) did not affect the acceleration of wound closure by indirubin. MTT assay and BrdU assay confirmed that indirubin does not promote the proliferation of keratinocytes (Fig. 2C,D). In contrast, when the cells were treated with cytochalasin D, an inhibitor of cell migration, wound closure was markedly inhibited regardless of the presence of indirubin (Fig. 2E). Thus, the acceleration of wound closure by indirubin probably occurs through the promotion of cell migration.

### Indirubin-induced cell migration/wound closure is independent of AHR

**Figure 3:**
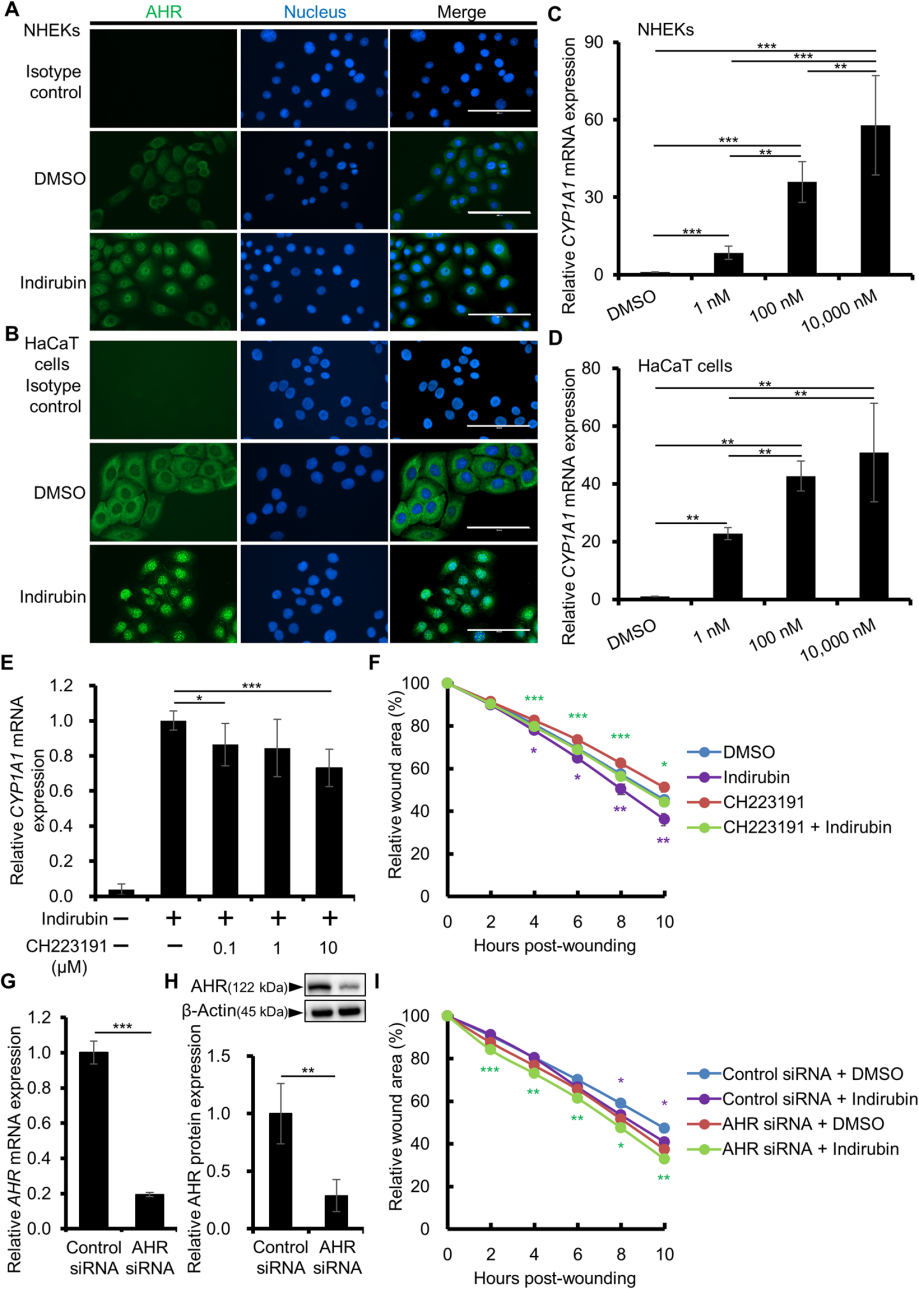
Indirubin promotes keratinocyte migration in an AHR-independent manner. (**A,B**) NHEKs (**A**) or HaCaT cells (**B**) were treated with DMSO (0.1%) or indirubin (100 nM) for 3 or 6 h and the nuclear translocation of AHR was assessed by immunocytochemistry. Representative images are shown. Scale bar = 100 μm. (**C,D**) NHEKs (**C**) or HaCaT cells (**D**) were treated with DMSO (0.1%) or indirubin (1, 100, or 10,000 nM) for 6 h and assessed for *CYP1A1* expression (n = 6). (**E**) HaCaT cells were treated with DMSO (0.1%) or indirubin (100 nM) in the absence or presence of an AHR antagonist, CH223191 (0.1, 1, or 10 μM), for 6 h and assessed for *CYP1A1* expression (n = 6). (**F**) HaCaT cells were scratched and treated with DMSO (0.1%) or indirubin (100 nM) in the absence or presence of CH223191 (10 μM). Relative wound areas are shown (n = 18) and the statistical significances between DMSO and indirubin or CH223191 and CH223191 + indirubin are indicated with purple or green asterisks, respectively. (**G**,**H**) HaCaT cells were transfected with control or AHR siRNA and knockdown efficiency was evaluated at (**G**) *AHR* mRNA and (**H**) AHR protein levels (n = 3). Cropped blots are displayed above the graph and full-length blots are shown in Supplementary Fig. S2. The samples derive from the same experiment and that blots were processed in parallel. (**I**) The siRNA-transfected cells were scratched and treated with DMSO (0.1%) or indirubin (100 nM). Relative wound areas are shown (n = 12) and the statistical significances between DMSO and indirubin in control siRNA transfected cells or AHR siRNA transfected cells are indicated with purple or green asterisks, respectively. Data are presented as mean ± SD (**C–G**,**I**) or SEM (**H**). *P < 0.05, **P < 0.01, and ***P < 0.001.

We next examined whether indirubin activates the AHR/CYP1A1 axis. Indirubin induced the cytoplasmic-to-nuclear translocation of AHR (Fig. 3A,B) and upregulated *CYP1A1* expression (Fig. 3C,D) in normal human epidermal keratinocytes (NHEKs) as well as in HaCaT keratinocyte cell line. To investigate the role of AHR in the indirubin-induced upregulation of migration/wound closure, we first inhibited AHR of keratinocytes using its specific antagonist, CH223191. CH223191 inhibited the indirubin-induced *CYP1A1* expression (Fig. 3E). However, it did not inhibit the indirubin-induced wound closure (Fig. 3F). To confirm this, we knocked down AHR using AHR siRNA. The efficiency of AHR knockdown was 80.50 ± 0.69% at the mRNA level (Fig. 3G) and 77.33 ± 12.90% at the protein level (Figs. 3H, S2). In accordance with the result of CH223191 treatment, AHR knockdown by siRNA did not cancel the accelerated wound closure in the presence of indirubin (Fig. 3I). These results indicated that indirubin promotes wound closure in an AHR-independent manner.

### Indirubin-induced cell migration/wound closure is dependent on PXR

**Figure 4:**
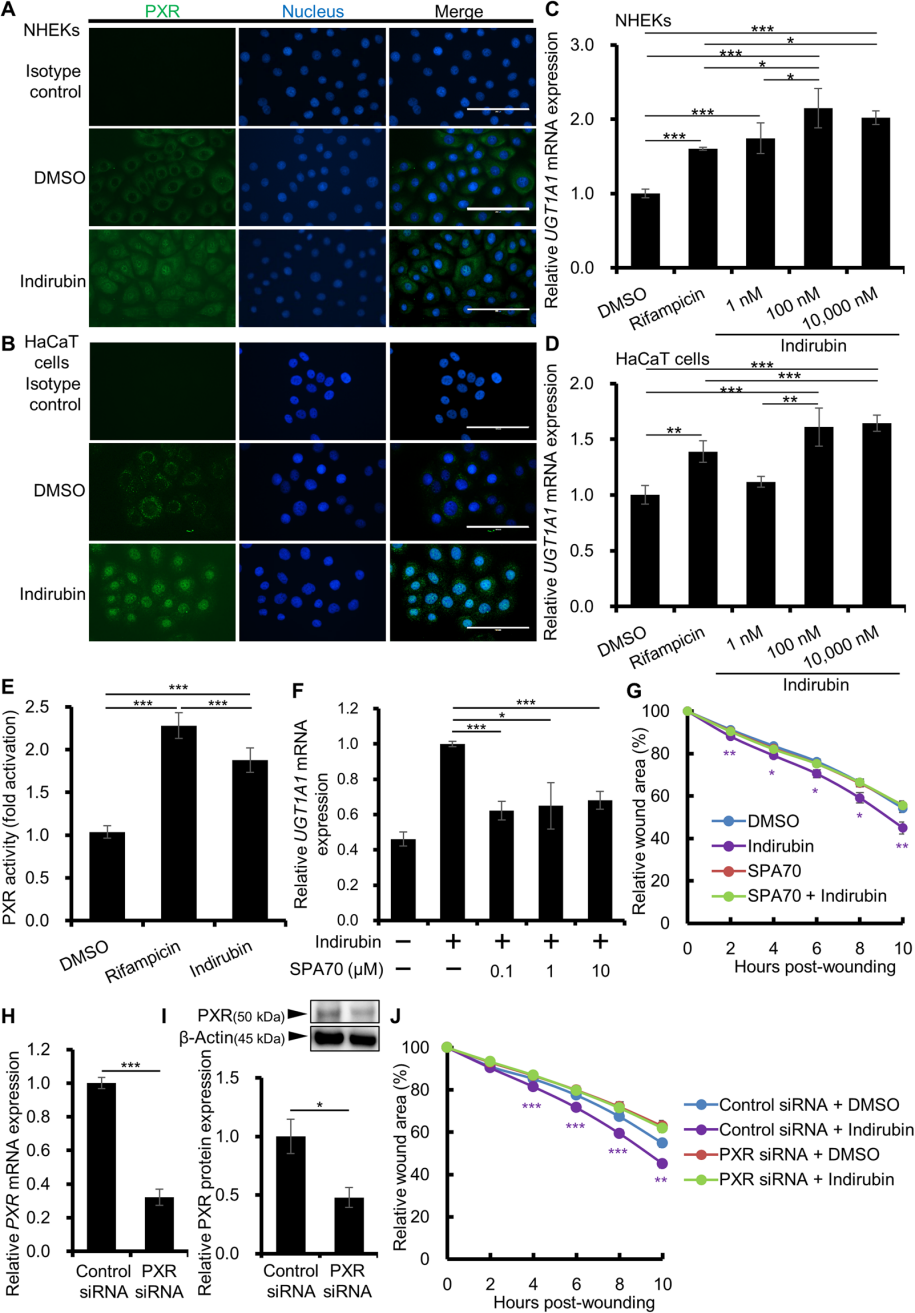
Indirubin activates PXR and promotes wound healing through PXR. (**A,B**) NHEKs (**A**) or HaCaT cells (**B**) were treated with DMSO (0.1%) or indirubin (100 nM) for 6 h and the nuclear translocation of PXR was assessed by immunocytochemistry. Representative images are shown. Scale bar = 100 μm. (**C,D**) NHEKs (**C**) or HaCaT cells (**D**) were treated with DMSO (0.1%) or indirubin (1, 100, or 10,000 nM) for 6 h and assessed for *UGT1A1* expression (n = 6). (**E**) Cells were treated with DMSO (0.1%), indirubin (100 nM), or rifampicin (100 nM) for 48 h and PXR activation was assessed by luciferase reporter assay. Reactions were performed in triplicate wells and PXR activation was calculated by dividing normalized luciferase activity of indirubin-treated condition by that of DMSO-treated control and was shown as fold activation relative to the vehicle control. (**F**) HaCaT cells were treated with DMSO (0.1%) or indirubin (100 nM) in the absence or presence of a PXR antagonist, SPA70 (0.1, 1, or 10 μM), for 6 h and assessed for *UGT1A1* expression (n = 6). (**G**) HaCaT cells were scratched and treated with DMSO (0.1%) or indirubin (100 nM) in the absence or presence of SPA70 (10 μM). Relative wound areas are shown (n = 18) and the statistical significances between DMSO and indirubin are indicated with purple asterisks. (**H,I**) HaCaT cells were transfected with control or PXR siRNA and knockdown efficiency was evaluated by measuring (**H**) *PXR* mRNA and (**I**) PXR protein (n = 3). Cropped blots are displayed above the graph and full-length blots are shown in Supplementary Fig. S5. The samples derive from the same experiment and that blots were processed in parallel. (**J**) The siRNA-transfected cells were scratched and treated with DMSO (0.1%) or indirubin (100 nM). Relative wound areas are shown (n = 12) and the statistical significances between DMSO and indirubin in control siRNA transfected cells are indicated with purple asterisks. Data are presented as mean ± SD (**C–H, J**) or SEM (**I**). *P < 0.05, **P < 0.01, and ***P < 0.001.

Since the effect of indirubin was independent of AHR, we next examined the involvement of PXR. As shown in Fig. 4A,B, indirubin activated PXR and induced its cytoplasmic-to-nuclear translocation in keratinocytes. To confirm the activation of PXR by indirubin, the expression of *UGT1A1*, one of the downstream genes of PXR, was assessed. A well-known PXR agonist, rifampicin, was used as a positive control. *UGT1A1* was significantly upregulated by indirubin in both NHEKs (Fig. 4C) and HaCaT cells (Fig. 4D). Another PXR target gene *CYP3A4* was also assessed and as shown in Supplementary Fig. S3A, rifampicin slightly but significantly upregulated the *CYP3A4* expression in NHEKs. Indirubin also tended to slightly increase the expression of *CYP3A4*, but the differences were not statistically significant at concentrations of 1 and 100 nM (Supplementary Fig. S3A). However, when NHEKs were scratched before indirubin treatment, the *CYP3A4* expression was significantly upregulated compared with that of DMSO- or indirubin-treated unscratched cells or DMSO-treated scratched cells (Supplementary Fig. S3B), implying that indirubin and scratching synergistically activate PXR. In the scratch assay, indirubin (100 nM) accelerated the wound closure in NHEKs similarly to that in HaCaT cells (Supplementary Fig. S4). We further confirmed the activation of PXR by indirubin using luciferase reporter assay. As shown in Fig. 4E, indirubin (100 nM)-treatment induced significant activation of PXR compared to that of DMSO-treated control condition (1.88 ± 0.14-fold increase compared to control), confirming the activation of PXR by indirubin in our assay system.

We next investigated the effect of PXR inhibition on the indirubin-induced wound closure. A PXR specific antagonist SPA70 was used and it inhibited the indirubin-induced *UGT1A1* expression (Fig. 4F). When the scratched cells were treated with both SAP70 and indirubin, indirubin-induced wound closure was completely inhibited (Fig. 4G). To confirm this, we knocked down PXR using PXR siRNA. The efficiency of PXR knockdown was 67.83 ± 2.75% at the mRNA level (Fig. 4H) and 52.18 ± 8.55% at the protein level (Figs. 4I, S5). Unlike AHR knockdown, PXR knockdown entirely abrogated the indirubin-induced promotion of wound closure (Fig. 4J), implying that indirubin promotes wound closure in a PXR-dependent manner.

### Indirubin promotes phosphorylation of JNK and wound closure through PXR

**Figure 5:**
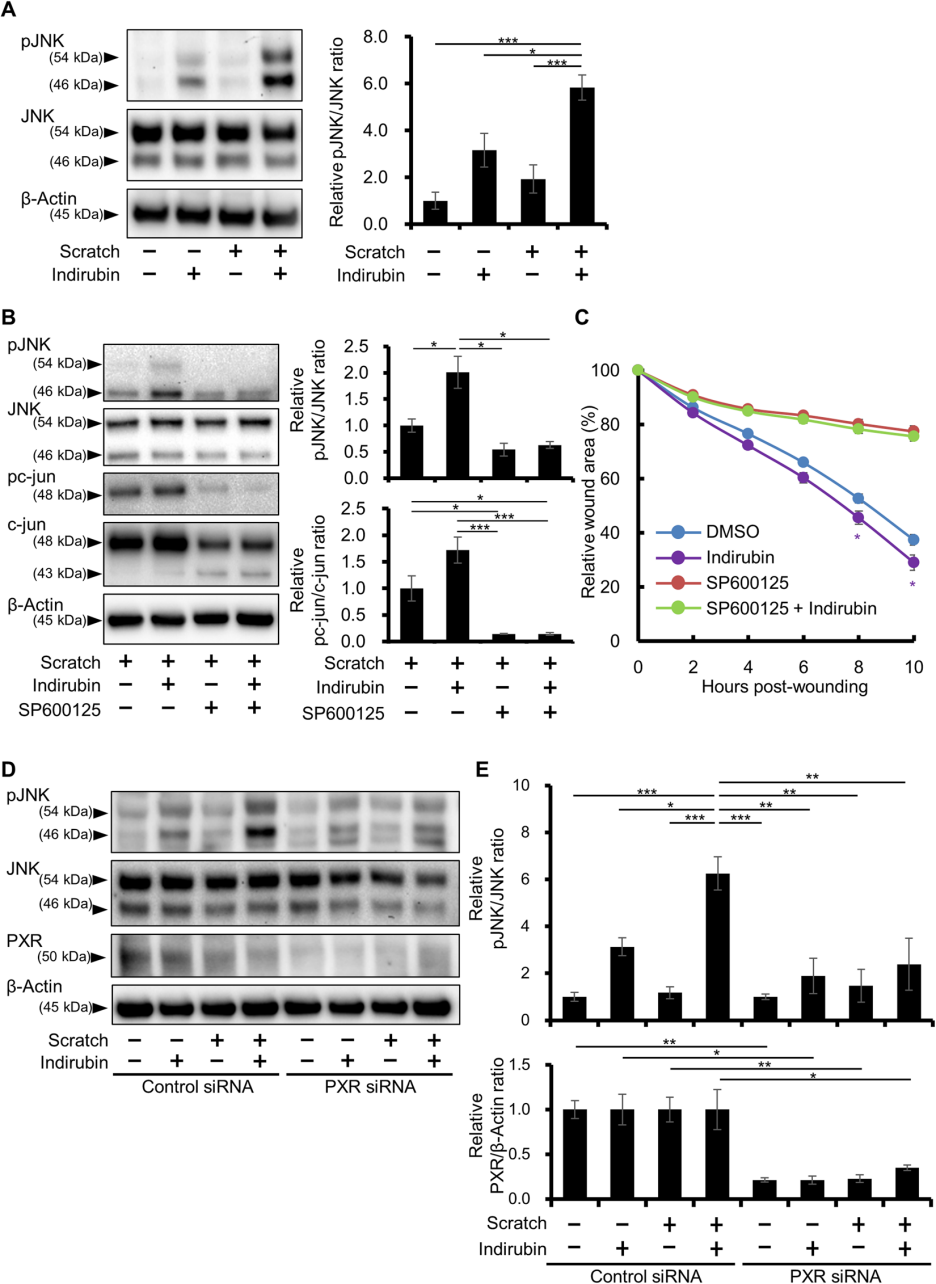
Indirubin promotes wound healing via the activation of JNK signaling though PXR. (**A**) Unscratched or scratched HaCaT cells were further treated with DMSO (0.1%) or indirubin (100 nM) for 6 h and assessed for the phosphorylation of JNK. Representative images (left) and relative pJNK/JNK ratio (right) are shown (n = 3). Cropped blots are displayed and full-length blots are shown in Supplementary Fig. S6. (**B,C**) HaCaT cells were scratched and treated with DMSO (0.1%) or indirubin (100 nM) in the absence or presence of JNK inhibitor SP600125 (40 μM). Representative images (**B**, left), relative pJNK/JNK and pc-jun/c-jun ratios measured by western blotting (**B**, right, n = 3), and relative wound areas (**C**, n = 18) are shown. The statistical significances between DMSO and indirubin in the absence of SP600125 are indicated with purple asterisks in (**C**). Cropped blots are displayed and full-length blots are shown in Supplementary Fig. S8. (**D,E**) HaCaT cells were transfected with control or PXR siRNA. The transfected cells were then scratched or left unscratched, and were further treated with DMSO (0.1%) or indirubin (100 nM). At 6 h post-treatment, cells were harvested and the phosphorylation status of JNK was assessed by western blotting. Representative images (**D**), the relative pJNK/JNK ratio (**E**, upper), and the relative PXR/β-Actin ratio (**E**, lower) are shown (n = 3). Cropped blots are displayed and full-length blots are shown in Supplementary Fig. S9. The samples derive from the same experiment and that blots were processed in parallel for (**A,B,D**), respectively. Data are presented as mean ± SD (**C**) or SEM (**A,B,E**). *P < 0.05, **P < 0.01, and ***P < 0.001.

To obtain further insight into the mechanisms underlying the indirubin-promoted wound closure, the phosphorylation of cell migration-related signaling molecules was evaluated. Among the molecules tested, only the phosphorylation of JNK was significantly increased at 5.83 ± 1.32-fold in indirubin- and scratch-cotreated cells compared with that in DMSO-treated, unscratched cells (Figs. 5A, S6). Single treatment with indirubin or scratching tended to increase the phosphorylation of JNK (3.16 ± 1.74-fold and 1.92 ± 1.47-fold, respectively); however, the difference was not statistically significant (Fig. 5A). The phosphorylation of Akt and ERK was not increased by indirubin, scratch, or the combination of indirubin and scratch (Supplementary Fig. S7). To assess the involvement of JNK signaling in the indirubin-induced wound closure, JNK was inhibited with an inhibitor specific for it, SP600125. In the presence of SP600125, the phosphorylation of JNK and its downstream c-jun was significantly prevented irrespective of the presence of indirubin in the scratched keratinocytes (Figs. 5B, S8). In line with these results, the indirubin-induced acceleration of wound closure was canceled in the presence of SP600125 (Fig. 5C), indicating that indirubin promotes cell migration and wound closure through the activation of JNK signaling in the scratched condition.

To elucidate the involvement of PXR in indirubin-induced JNK activation, PXR was knocked down with siRNA and the phosphorylation of JNK was assessed by western blotting. As shown in Fig. 5D,E, PXR was downregulated by siRNA transfection confirmed by western blotting (knockdown efficiency 75.03 ± 8.14%) and the knockdown of PXR significantly inhibited the JNK phosphorylation induced by indirubin and scratch (Figs. 5D,E, and S9). Taking these findings together, it is proposed that indirubin-induced promotion of keratinocyte migration and wound healing is mediated via the PXR-JNK axis.

### PXR is expressed in keratinocytes of human skin ulcer

**Figure 6:**
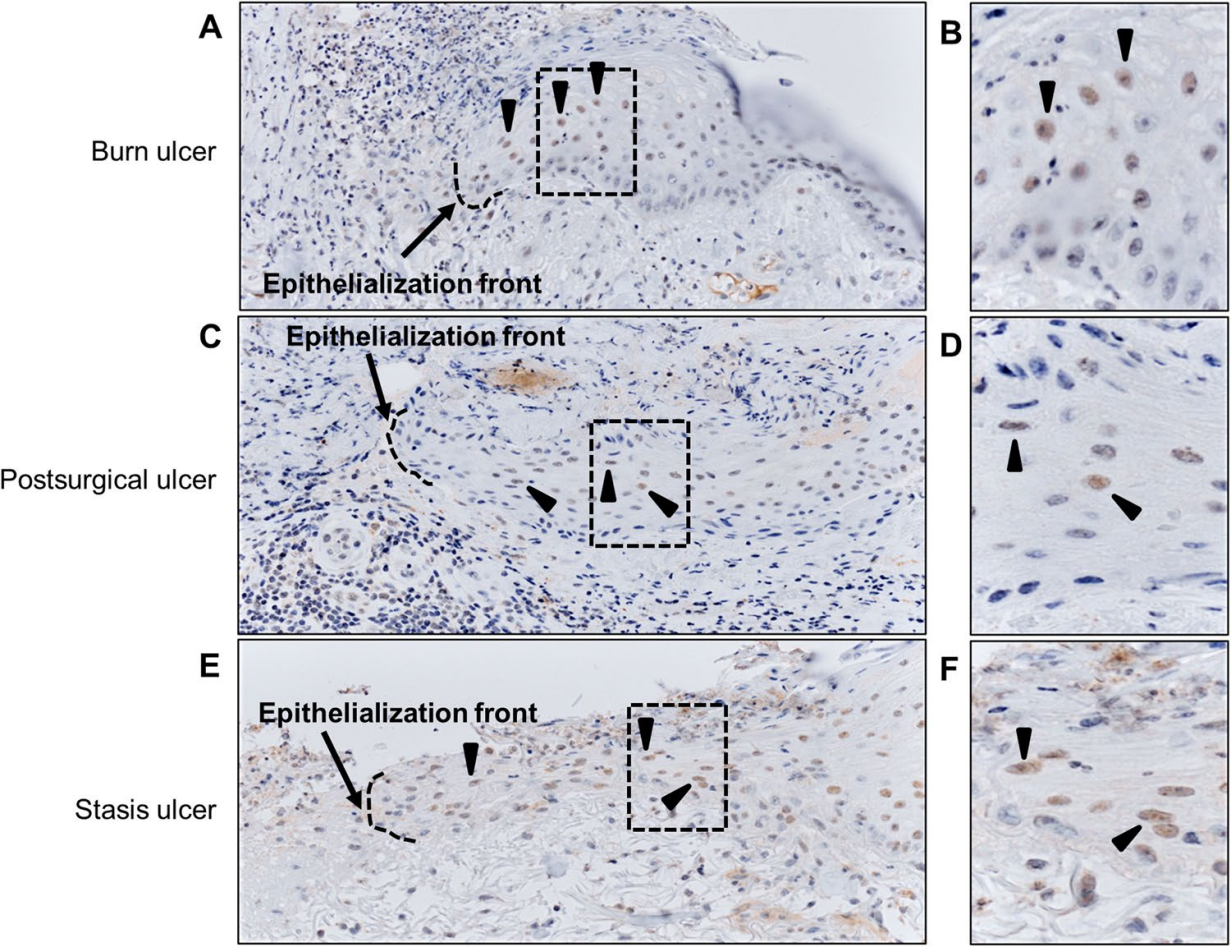
PXR expression in human skin ulcer. Human skin ulcer tissues were stained for PXR by immunohistochemistry. Representative images of (**A,B**) burn ulcer, (**C,D**) postsurgical ulcer, and (**E,F**) stasis ulcer are shown. Broken line and arrow indicate epithelization front and arrowheads indicate PXR expressed in the nuclei of keratinocytes in the re-epithelialized islands. Magnified images of the areas boxed with a broken line in (**A,C,D**) are shown as (**B,D,F**), respectively.

Finally, we assessed the immunohistological localization of PXR in human skin ulcers, namely, burn, postsurgical, and stasis ulcers. Regardless of the ulcer type, PXR was detected in the nuclei of keratinocytes (indicated by an arrowhead) in the re-epithelialized islands from the epithelization front to its base (Fig. 6).

## Discussion

Among various beneficial phytochemicals, indigo naturalis extract and its salubrious component indirubin have drawn particular attention because they have been shown to exert potent anti-inflammatory effects in mouse models of inflammatory epithelial disorders, such as psoriasis and ulcerative colitis^7,14,25,26^. In these mouse models, indirubin inhibits the proliferation and accelerates the differentiation of keratinocytes^7^ and induces Foxp3-expressing regulatory T cells^26^. These diverse protective and anti-inflammatory effects of indirubin are believed to be mediated by the xenobiotic chemical receptor and transcription factor AHR^14^. The binding of ligands activates AHR, further accelerates epidermal terminal differentiation, and protects skin barrier function^27,28^. AHR activation also contributes to the maintenance of intestinal barrier integrity^29^ and, consistent with this, experimental colitis is much severer in *Ahr*-deficient mice than in their wild-type counterparts^30^. Additionally, AHR activation preferentially promotes regulatory T cells in the gut, which is protective against T-cell-mediated colitis^31^.

In this study, we demonstrated that indirubin facilitated keratinocyte migration *in vitro* and accelerated wound closure *in vivo*. Indirubin activated AHR and induced its cytoplasmic-to-nuclear translocation with subsequent *CYP1A1* upregulation. However, the indirubin-induced migratory capacity of keratinocytes was independent of AHR since the effect of indirubin was not canceled by the knockdown of AHR or by the administration of an AHR-specific inhibitor. As a previous study indicated the possibility that indirubin activates another xenobiotic chemical receptor, PXR^17^, we next examined whether indirubin activates PXR in our assay system. Similar to its effects on AHR, indirubin activated PXR and induced its cytoplasmic-to-nuclear translocation. Using luciferase reporter assay, PXR activation by indirubin (100 nM) was further confirmed. In general, the activation of PXR upregulates the expression of the downstream genes, such as *UGT1A1*^23,24^ and *CYP3A4*^17,32,33^. The expression of *UGT1A1* was significantly induced by indirubin and it was impaired by treatment with PXR antagonist SPA70, indicating that indirubin does activate PXR and induce its downstream gene *UGT1A1* in keratinocytes. On the other hand, although *CYP3A4* is one of the major target gene of PXR, keratinocytes are known to be poor producers of this xenobiotic-catabolizing enzyme^34–36^. As expected, the indirubin-induced PXR activation did not upregulate *CYP3A4* expression in non-scratched keratinocytes. However, notably, indirubin significantly augmented *CYP3A4* expression when the keratinocytes were scratched, indicating that indirubin and scratch synergistically activate PXR. In accordance with the activation of the PXR by indirubin and scratching, the transfection of PXR siRNA or inhibition of PXR by SPA70 significantly abrogated the indirubin-induced keratinocyte migration in the scratch assay. In a previous study using PXR-overexpressing human hepatocellular carcinoma HepG2 cells, it was proposed that PXR activation induces morphological change and migration of the cells^33^. Another study using HepG2 cells also indicated that PXR promotes the migration of these cells^37^. Noteworthy, wound healing effect of indirubin was observed at 100 nM but not at higher (10,000 nM) concentration. As far as we know, it is reported that the higher concentration i.e. at μM level of indirubin or its derivatives inhibits the invasion and migration of tumor-derived cells *in vitro*^38,39^. Therefore we assume that higher concentration of indirubin negatively affects wound healing (so called high dose inhibition) and 100 nM was the optimum concentration in our experimental condition in keratinocytes. Taking these findings together, it is suggested that indirubin facilitates keratinocyte migration and wound healing via PXR activation, but not AHR signal.

To the best of our knowledge, the present study is the first to show the PXR-mediated acceleration of cutaneous wound healing by indirubin. Although the other PXR agonist rifampicin reportedly accelerates wound closure in intestinal epithelia^18^, it did not promote the wound closure in keratinocytes (Supplementary Fig. S10). Thus, it seems that the acceleration of wound healing through PXR is specific to the activation with indirubin in keratinocytes. This fact raises the possibility of the involvement of additional factor in indirubin-induced wound healing of keratinocytes and it will be addressed in the future study. In addition to nutritional uptake from herbs and vegetables, indirubin is also biosynthesized from tryptophan by commensal microbiota such as *Escherichia coli* in the intestine and *Malassezia* yeast in the skin^15,40,41^. Evidence has suggested that the intestinal indirubin is absorbed into the blood, binds to serum albumin, circulates throughout the body, and is excreted into the urine^10,42,43^. This circulating indirubin exerts the biological function of activating the AHR pathway, among other potential functions^10^. Although indirubin is a dual agonist for AHR and PXR, our results clearly revealed that the PXR activation is crucial for wound closure, rather than that of AHR. In accordance with this, nuclear PXR was clearly observed in the re-epithelializing keratinocytes comprising the epithelization islands of human ulcers.

JNK signaling plays a pivotal role in epithelial wound healing by facilitating the migration and proliferation of keratinocytes^44,45^. JNK is also known to promote the migration of HaCaT keratinocytes through activation of the PI3K/AKT and JNK pathways^46^. In the present scratch model, indirubin induced the phosphorylation of JNK and the JNK inhibitor SP600125 abrogated the indirubin-induced acceleration of wound closure. Notably, PXR-mediated CYP3A4 upregulation is also reported to be JNK-dependent^47^, suggesting the importance of the PXR-JNK axis in the process of wound closure. In conclusion, indirubin is a potential endogenous intestinal chemical that is beneficial to the skin by facilitating cutaneous wound closure via PXR activation.

## Methods

### Reagents and antibodies

Indirubin (purity more than 98%, Sigma-Aldrich, St. Louis, MO) was dissolved in dimethyl sulfoxide (DMSO; Sigma-Aldrich) and added to culture medium at final concentrations ranging from 1 nM to 10 μM. The AHR antagonist CH223191 (Merck, Darmstadt, Germany), the PXR antagonist SPA70 (Axon Medchem, Groningen, The Netherlands) the PXR agonist rifampicin (Sigma-Aldrich), the JNK inhibitor SP600125 (Abcam, Cambridge, UK), and the inhibitor of actin polymerization cytochalashin D (Sigma-Aldrich) were dissolved in DMSO and added to culture medium at final concentrations of 10, 10, 10, 40, and 2 μM, respectively. MMC (Roche, Basel, Switzerland) was dissolved in distilled water and used at a final concentration of 5 μg/mL. The antibodies used were as follows: rabbit anti-human β-actin antibody, rabbit anti-phosphorylated ERK1/2 (Thr202/Tyr204), rabbit anti-ERK1/2, rabbit anti-phosphorylated Akt (Ser473), rabbit anti-Akt, rabbit anti-phosphorylated JNK (Thr183/Tyr185), rabbit anti-JNK, rabbit anti-phosphorylated c-jun, and rabbit anti-c-jun antibody (all purchased from Cell Signaling Technology, Danvers, MA), rabbit anti-human AHR antibody (H-211) (Santa Cruz Biotechnology, Dallas, TX), mouse anti-human PXR antibody (Abcam), rabbit anti-human PXR antibody (Thermo Fisher Scientific, Waltham, MA) and HRP-conjugated anti-rabbit or anti-mouse secondary antibody (Cell Signaling Technology). For immunostaining, AlexaFluor488^®^-conjugated anti-rabbit or anti-mouse secondary antibodies (Thermo Fisher Scientific) were used.

### Cell culture

HaCaT cells (Cell Lines Service, Eppelheim, Germany), an immortalized human keratinocyte cell line, were maintained in Dulbecco’S Modified Eagle’s Medium (DMEM; Sigma-Aldrich) supplemented with 10% FBS (Sigma-Aldrich), 100 units/mL penicillin, and 100 μg/mL streptomycin (Thermo Fisher Scientific). Cells were passaged every 2 to 3 days at sub-confluence. NHEKs (Lonza, Basel, Switzerland) were maintained in KBM-Gold^TM^ Keratinocyte Basal Medium supplemented with KGM-Gold^TM^ Keratinocyte Growth Medium SingleQuots^TM^ Supplements and Growth Factors (Lonza), and the medium was replaced every other day. NHEKs were passaged at sub-confluence and used for experiments.

### Immunocytochemistry

Cells were seeded on an eight-well μ-Slide (ibidi GmbH, Martinsried, Germany) at a density of 1,000 cells per well and incubated for 48 h. Cells were treated with DMSO (0.1%) or indirubin (100 nM) for 3 or 6 h, and then fixed with cold acetone, air-dried, and treated with 5% bovine serum albumin (Sigma-Aldrich) to block the non-specific binding of antibodies. After washing with Dulbecco’s PBS (DPBS), fixed cells were incubated with primary antibodies at 4 °C overnight and further treated with AlexaFluor488^®^-conjugated secondary antibodies. Cells were then covered with Vectashield mounting medium with DAPI (Vector Laboratories, Burlingame, CA) and observed by EVOS^®^ FL fluorescent microscopy (Thermo Fisher Scientific).

### Immunohistochemistry

Skin ulcer tissue samples were obtained from three patients (burn ulcer, postsurgical ulcer, and stasis ulcer). Informed consent was obtained from each patient. Paraffin-embedded tissues were sectioned at a thickness of 3 μm and stained for PXR using Histofine Simple Stain Max-PO (Multi) (Nichirei Biosciences Inc., Tokyo, Japan) and the automated staining device Histostainer (Nichirei Biosciences Inc.). Hematoxylin was used for the counterstaining of nuclei. Images were captured using a virtual slide system and OlyVIA software (Olympus, Tokyo, Japan). This study was approved by the Institutional Ethics Committee of Kyushu University Hospital (Approval ID: 30-363), and conducted in accordance with the Declaration of Helsinki. Written informed consent was received from the patients prior to their inclusion in the study.

### Scratch assay

Cells were seeded at a density of 2 × 10^4^ cells per well of a 96-well ImageLock tissue culture microplate (Essen Bioscience, Ann Arbor, MI) pre-coated with type I collagen (Nitta Gelatin Inc., Osaka, Japan). At full confluence, cell monolayers were scratched with a wound-maker (Essen Bioscience) and the scratched cells were incubated with DMSO (0.1%), indirubin (1, 10, 100, or 10,000 nM), or various inhibitors in DMEM supplemented with 10% FBS. The wound area of each well was automatically imaged every 2 h in a CO_2_ incubator. The wound area relative to that at 0 h was measured using IncuCyte software (Essen Bioscience).

### Cell proliferation assay

The MTT assay (Trevigen, Gaithersburg, MD) and BrdU incorporation assay (Roche) were performed in accordance with the manufacturers’ instructions. For both assays, HaCaT cells were seeded in 96-well plates at a density of 5,000 cells per well and incubated for 24 h. Cells were then treated with DMSO (0.1%) or indirubin (1, 10, or 100 nM) for 24 h. For the BrdU assay, BrdU was applied in the final 2 h of indirubin treatment. The reaction products were quantified using a microplate reader (Bio-Rad Laboratories Inc., Hercules, CA) by measuring the absorbance at 570 nm for the MTT assay, or at 450 nm (reference wavelength 655 nm) for the BrdU incorporation assay.

### Quantitative reverse-transcription polymerase chain reaction (qRT-PCR)

Total RNA was extracted from the cells using RNeasy Mini Kit (Qiagen, Hilden, Germany) following the manufacturer’s instructions, converted to cDNA using PrimeScript RT Reagent Kit (TaKaRa Bio Inc., Kusatsu, Japan), and then applied for qPCR with SYBRGreen Premix Ex Taq (TaKaRa Bio Inc.). Reaction cycles were as follows: 95 °C for 30 s and then 40 cycles of 95 °C for 5 s and 60 °C for 20 s. The expression of each gene was normalized to the cycle threshold of β-actin (*ACTB*). Primers used for PCR are listed in Table 1.

**Table 1:** Primer sequences for qRT-PCR.

Gene Symbol		Sequence
*ACTB*	Sense	5′-ATTGCCGACAGGATGCAGA-3′
	Anti-sense	5′-GAGTACTTGCGCTCAGGAGGA-3′
*AHR*	Sense	5′-CAAATCCTTCCAAGCGGCATA-3′
	Anti-sense	5′-CGCTGAGCCTAAGAACTGAAAG-3′
*CYP1A1*	Sense	5′-TAGACACTGATCTGGCTGCAG-3′
	Anti-sense	5′-GGGAAGGCTCCATCAGCATC-3′
*CYP3A4*	Sense	5′-GAAACACAGATCCCCCTGAAA-3′
	Anti-sense	5′-ACTTACGGTGCCATCCCTTG-3′
*PXR*	Sense	5′-CCCAGCCTGCTCATAGGTTC-3′
	Anti-sense	5′-GGCGTAGCAAAGGGGTGTA-3′
*UGT1A1*	Sense	5′-CCTTGCCTCAGAATTCCTTC-3′
	Anti-sense	5′-ATTGATCCCAAAGAGAAAACCAC-3′

### siRNA transfection

Negative control siRNA, *AHR* siRNA (s1200), and *PXR* siRNA (s16911) (all purchased from Invitrogen, Carlsbad, CA) were each transfected into HaCaT cells using HiPerFect Transfection Reagent (Qiagen) or Lipofectamine RNAiMAX (Themo Fisher Scientific), in accordance with the manufacturers’ instructions. Cells were mixed with siRNA, incubated for 48 h, and used for scratch assay or western blotting. The knockdown efficiency was assessed by qRT-PCR and western blotting.

### Western blotting

HaCaT cells were seeded at a density of 3 × 10^5^ cells per well in six-well plates. At full confluence, cell monolayers were scratched by a blue pipette tip and treated with DMSO (0.1%) or indirubin (100 nM) for 6 h. Protein lysates of the cells were extracted with lysis buffer (25 mM HEPES, 10 mM Na_4_P_2_O_7_∙10H_2_O, 100 mM NaF, 5 mM EDTA, 2 mM Na_3_VO_4_, 1% Triton X-100) and analysed by SDS-PAGE on Bolt^TM^ 4–12% Bis-Tris Plus Gels (Invitrogen). Proteins were then transferred to PVDF membranes (Millipore), probed with specific primary antibodies, and further treated with HRP-conjugated secondary antibodies. Immunological bands were then visualized with SuperSignal West Pico Chemiluminescence Substrate (Thermo Fisher Scientific) and imaged using the ChemiDoc^TM^ XRS Plus System (Bio-Rad Laboratories Inc.). Signal of blots were analysed with Image Lab software (Bio-Rad Laboratories Inc.).

### Luciferase reporter assay

Luciferase reporter assay to evaluate PXR activation by indirubin was performed using Human pregnane X receptor activation assay system following the manufacturer’s instructions (Puracyp Inc., Carlsbad, CA). Briefly, cells were seeded into 96-well plate and incubated overnight at 37 °C in 5% CO_2_. Cells were then treated with DMSO (0.1%) or indirubin (100 nM). Rifampicin (100 nM) was used as positive control for PXR activation. Forty-eight hours post treatment, PXR activation was determined using ONE-Glo^TM^ Luciferase Assay System^TM^ and Celltiter-Fluor^TM^ (both from Promega, Madison, WI). Relative luminescence units (RLU) and relative fluorescence units (RFU) of each well were measured using EnSight Multimode Plate Reader (PerkinElmer Inc., Waltham, MA). PXR activation was then calculated by dividing normalized luciferase activity (RLU/RFU) of indirubin-treated condition by that of DMSO-treated control condition and was shown as fold activation relative to the vehicle control.

### Mice and wound healing assay *in vivo*

Six-week-old female BALB/c mice (Japan SLC, Inc., Shizuoka, Japan) were housed in a vivarium following the guidelines of the animal facility of Kyushu University. The mice were maintained on food and water ad libitum and handled in accordance with the Guidelines for the Care and Use of Laboratory Animals of Kyushu University. Wound healing assay *in vivo* was performed according to the method used in previous publication^48^. Mice were anesthetized with 2% to 3% sevoflurane and shaved, and then full-thickness wounds with a diameter of 6 mm were made on the dorsal skin using a biopsy punch (Kai Industries, Gifu, Japan). On the day of wounding and every 2 days thereafter, ointments of DMSO (1%)-containing Vaseline and indirubin (262.26 ng/g Vaseline, 1% DMSO)-containing Vaseline were applied to the wounds. Considering the difference between mono-layered keratinocytes and multiple-layered skin tissue, 10-times higher concentration of indirubin (1 μM) than *in vitro* (100 nM) experiment was used. Before applying these ointments, images of the wounds were taken until healing and the relative wound area was calculated from the images using ImageJ software (NIH, Bethesda, MD). All animal experiments were approved by the Animal Care and Use Committee, Kyushu University (Approval ID: A29-176-1).

### Statistics

Data are presented as mean ± SD or SEM. Statistical analyses were performed using GraphPad Prism7 software (GraphPad Software, San Diego, CA). The significance of differences between two groups was assessed by Student’s unpaired two-tailed *t*-test and that of three or more groups was assessed by one-way ANOVA followed by multiple comparisons. A P value less than 0.05 was considered to reflect statistical significance.

## Supplementary information

Supplementary Figure

## Data Availability

All data generated or analysed during this study are included in the main text and its Supplementary Information Files.
