# Supplementary material for: Indirubin-pregnane X receptor-JNK axis accelerates skin wound healing
Source: Sci Rep. 2019 Dec 3;9:18174. doi: 10.1038/s41598-019-54754-2 (PMC6890704; doi:10.1038/s41598-019-54754-2)
Supplement: Supplementary file 1 — Supplementary Figure [file 41598_2019_54754_MOESM1_ESM.pdf]

Indirubin-pregnane X receptor-JNK axis accelerates skin wound healing

Yuka Tanaka[1], Hiroshi Uchi[2], Takamichi Ito[1], and Masutaka Furue[1,3,4,*]

[1]Department of Dermatology, Graduate School of Medical Sciences, Kyushu University, Fukuoka 812-8582, Japan

[2]Department of Dermatology, National Hospital Organization Kyushu Cancer Center, Fukuoka 811-1395, Japan

[3]Research and Clinical Center for Yusho and Dioxin, Kyushu University Hospital, Fukuoka 812-8582, Japan

[4]Division of Skin Surface Sensing, Department of Dermatology, Graduate School of Medical Sciences, Kyushu University, Fukuoka 812-8582, Japan

*Corresponding author: Masutaka Furue, Department of Dermatology, Graduate School of Medical Sciences, Kyushu University, 3-1-1 Maidashi, Higashi-ku, Fukuoka City, Fukuoka 812-8582, Japan
Tel.: +81-92-642-5585, Fax: +81-92-642-5600
E-mail: furue@dermatol.med.kyushu-u.ac.jp

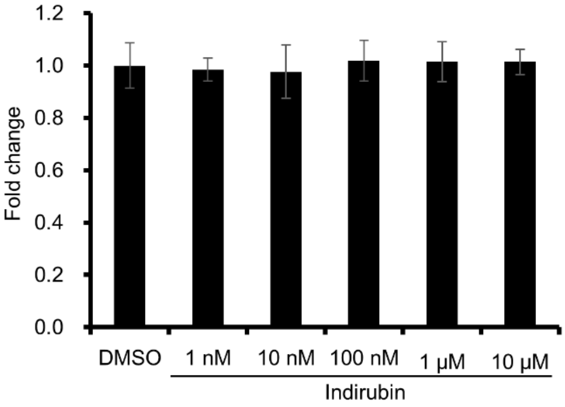

Supplementary Figure S1. Toxicity of indirubin on HaCaT keratinocytes.
HaCaT cells were treated with DMSO (0.1%) or indirubin (1, 10, 100 nM, or 1 or 10 μM) for 24 hours. The toxicity of indirubin was evaluated by a WST-8 formazan-based method. Data are presented as mean ± SD (n = 5 per group).

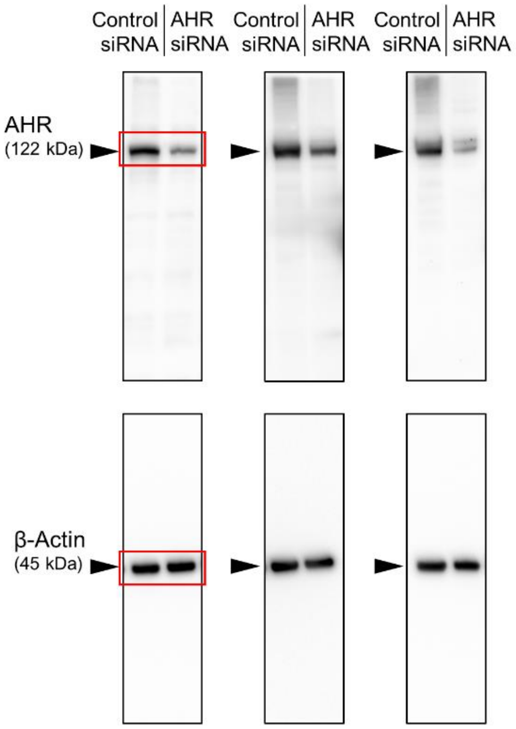

Supplementary Figure S2. Full length blots of AHR and β-Actin shown in Figure 3. HaCaT cells were transfected with control or AHR siRNA and knockdown efficiency was assessed by western blotting. Signal of AHR were analysed by Image Lab software and were normalized with the signal of β-Actin. The samples derive from the same experiment and that blots were processed in parallel. The gel images of individual replicate blots (n = 3) are shown and the image in Fig. 3H are indicated with the red boxes.

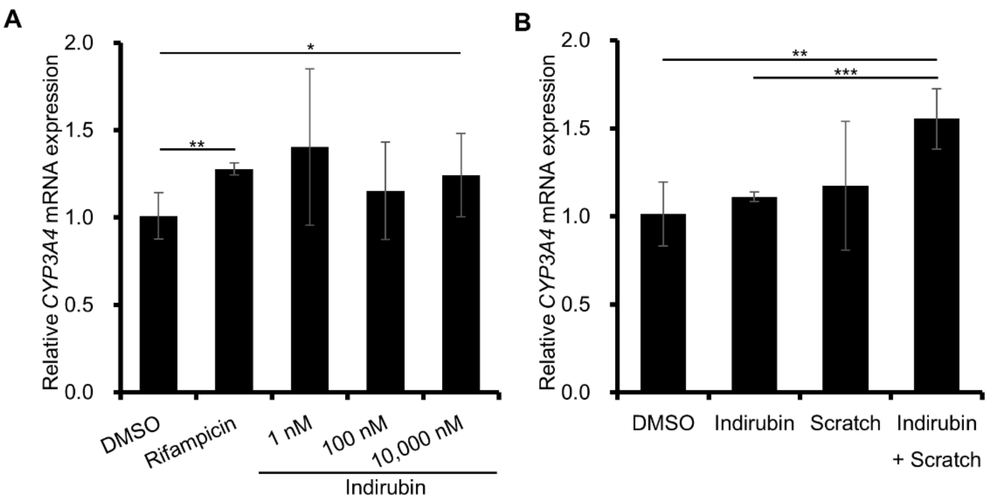

Supplementary Figure S3. Indirubin and scratching synergistically induce *CPY3A4* expression in NHEKs.

(A) NHEKs were treated with DMSO (0.1%) or indirubin (1, 100, or 10,000 nM) for 6 hours and assessed for the expression of *CYP3A4* mRNA by qPCR. Rifampicin (10 µM) served as a positive control of CYP3A4 induction. (B) Unscratched or scratched NHEKs were treated with DMSO (0.1%) or indirubin (100 nM) for 6 hours and assessed for the expression of *CYP3A4* mRNA by qPCR. Data are presented as mean ± SD (n = 3 per group). * $P < 0.05$, ** $P < 0.01$, and *** $P < 0.001$.

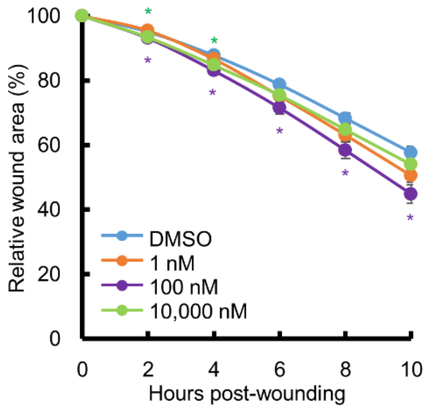

Supplementary Figure S4. Indirubin promotes wound closure in NHEKs *in vitro*. Monolayer NHEKs were scratched, treated with DMSO (0.1%) or indirubin (1, 100, or 10,000 nM), and assessed for relative wound areas. Data are presented as mean ± SD (n = 15–18 per condition). * $P < 0.05$.

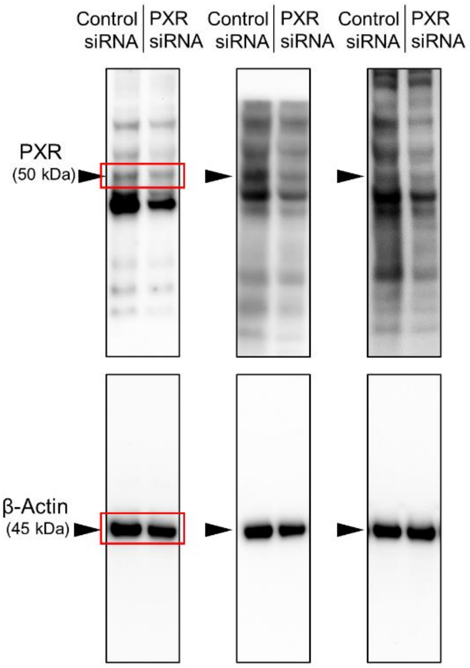

Supplementary Figure S5. Full length blots of AHR and β-Actin shown in Figure 4. HaCaT cells were transfected with control or PXR siRNA and knockdown efficiency was assessed by western blotting. Signal of PXR were analysed by Image Lab software and were normalized with the signal of β-Actin. The samples derive from the same experiment and that blots were processed in parallel. The gel images of individual replicate blots (n = 3) are shown and the image in Fig. 4I are indicated with the red boxes.

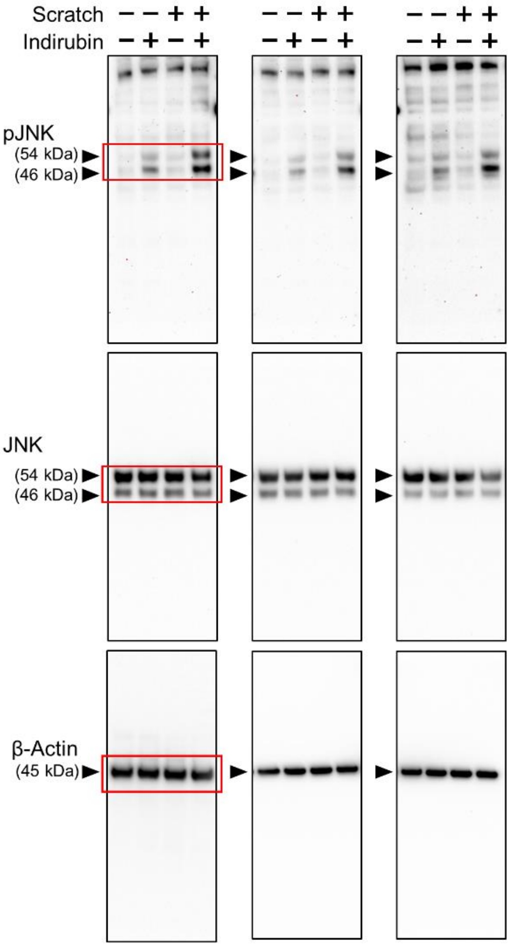

Supplementary Figure S6: Full length blots of JNK, pJNK, and β-Actin shown in Figure 5A.

The phosphorylation status of JNK was assessed by western blotting. Signals of each blots were analysed using Image Lab software. The samples derive from the same experiment and that blots were processed in parallel. Full length gel images of individual replicate blots (n = 3) are shown and the image in Fig. 5A are indicated with the red boxes.

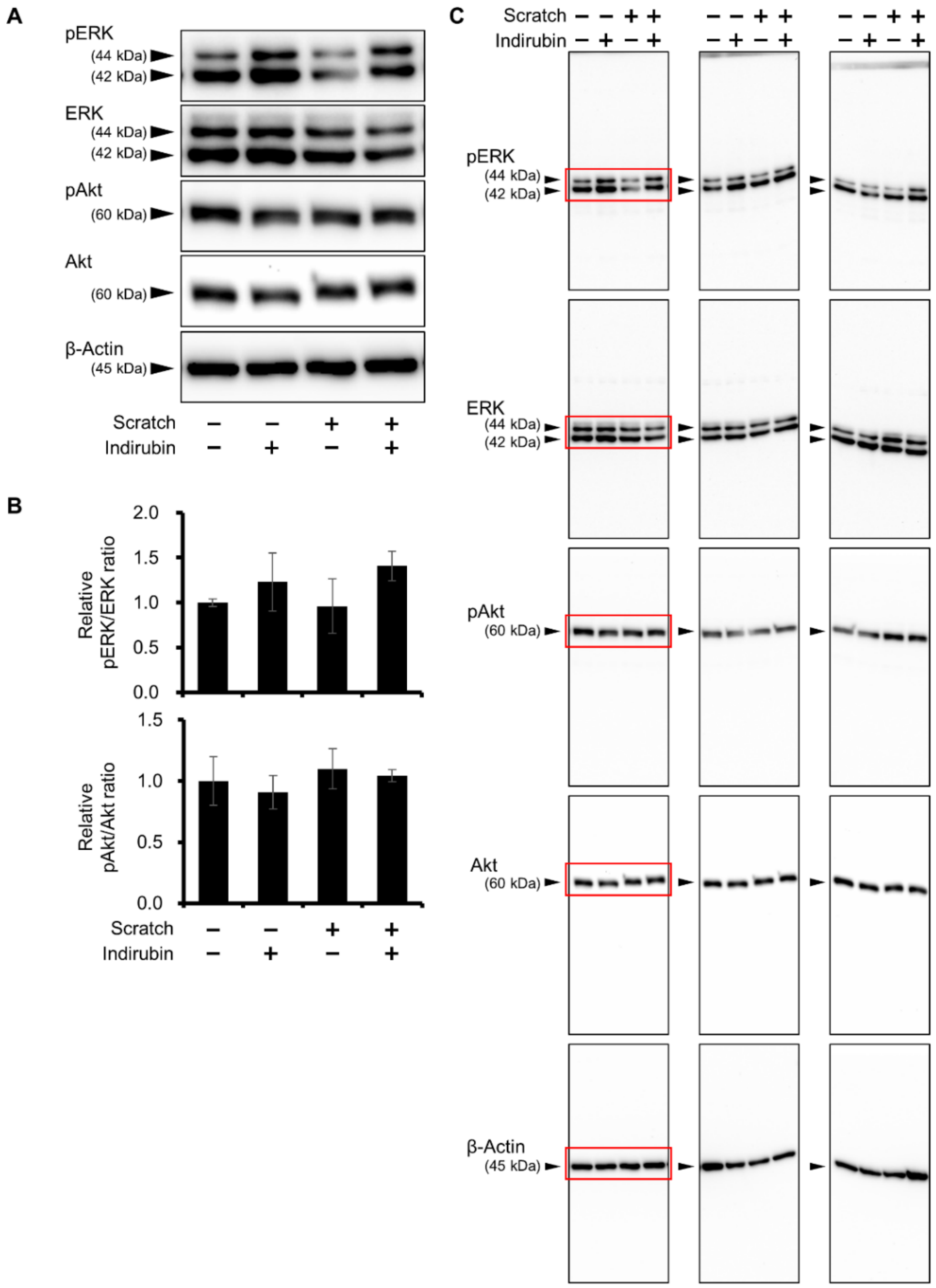

Supplementary Figure S7: ERK and Akt are not activated by indirubin or scratch. Unscratched or scratched HaCaT cells were incubated with or without indirubin for 6 h and assessed for the phosphorylation of ERK and Akt by western blotting. Representative images of immunoblotting (A) and the ratios of pERK/ERK and pAkt/Akt (B) are shown. Gel images were cropped and full-length blots are shown in Supplementary Fig. S7C. (C) Full length gel images of individual replicate blots (n = 3) are shown and the image in (A) are indicated with the red boxes. The samples derive from the same experiment and that blots were processed in parallel. Data are presented as mean ± SEM (n = 3).

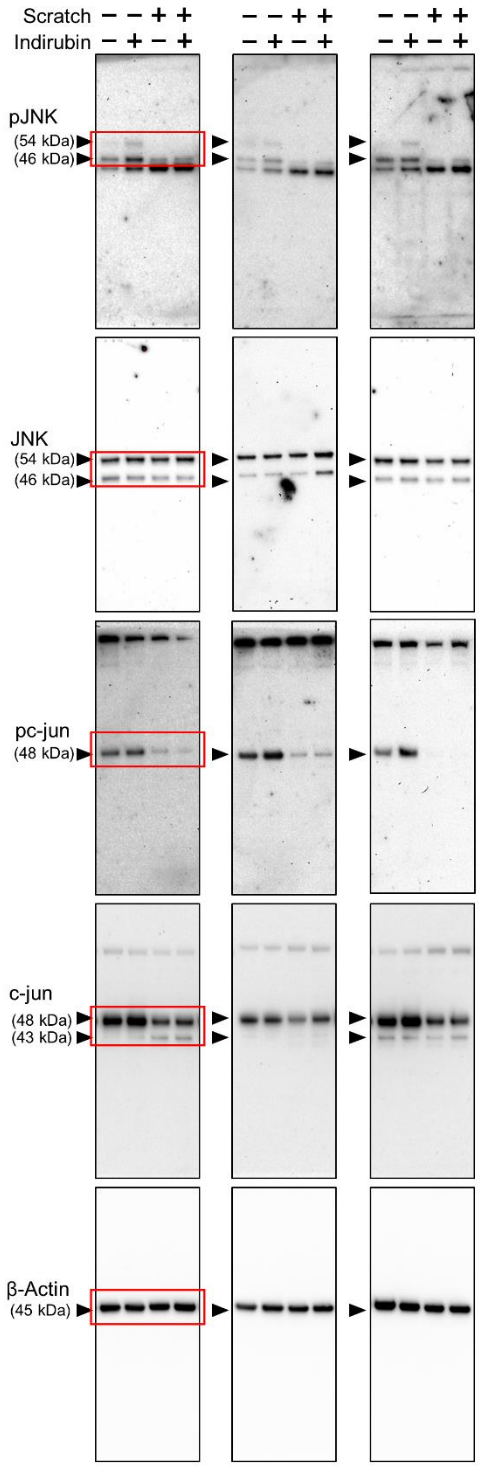

Supplementary Figure S8: Full length blots of JNK, pJNK, c-jun, and pc-jun, and β-Actin shown in Figure 5B.

The phosphorylation status of JNK and c-jun were assessed by western blotting. Signals of each blots were analysed using Image Lab software. The samples derive from the same experiment and that blots were processed in parallel. Full length gel images of individual replicate blots (n = 3) are shown and the image in Fig. 5B are indicated with the red boxes.

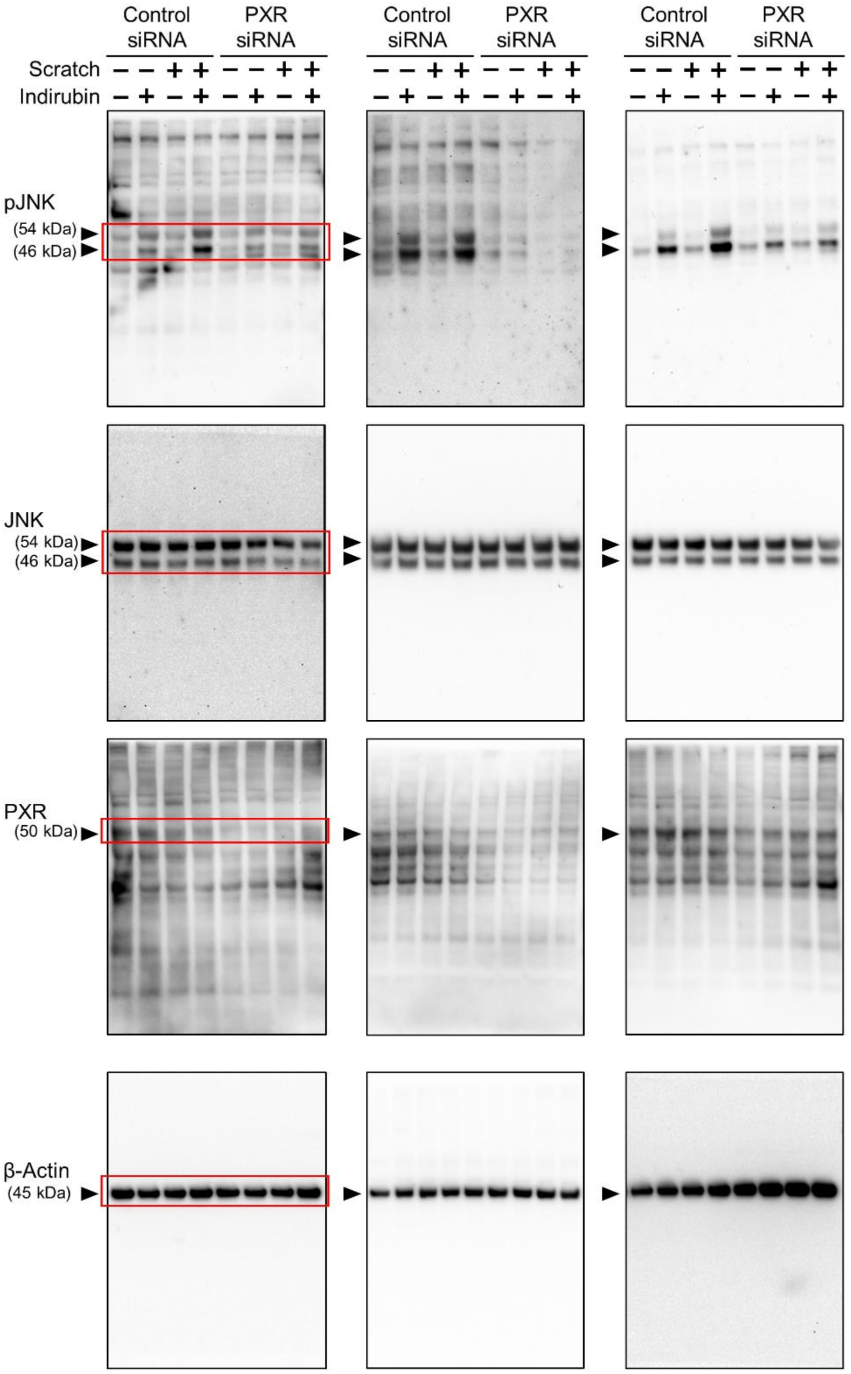

Supplementary Figure S9: Full length blots of JNK, pJNK, PXR, and β-Actin shown in Figure 5D.

The phosphorylation status of JNK and PXR expression were assessed by western blotting. Signals of each blots were analysed using Image Lab software. The samples derive from the same experiment and that blots were processed in parallel. Full length gel images of individual replicate blots (n = 3) are shown and the image in Fig. 5D are indicated with the red boxes.

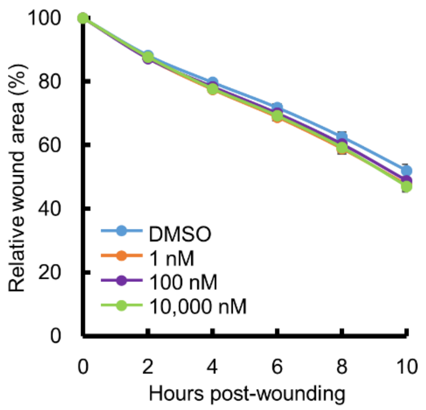

Supplementary Figure S10: Rifampicin does not promote wound closure in keratinocytes *in vitro*.

Monolayer HaCaT cells were scratched, treated with DMSO (0.1%) or rifampicin (1, 100, or 10,000 nM), and assessed for relative wound areas. Data are presented as mean ± SD (n = 18 per condition).
